# Surgeon and trainee perspectives on intraoperative education: a systematic review and meta-synthesis

**DOI:** 10.1080/10872981.2025.2560628

**Published:** 2025-09-24

**Authors:** Isaac Vaughn Ealing, Sarah Whereat, Rowena Forsyth, Jonathan Hong, Jerome Laurence

**Affiliations:** a Faculty of Medicine and Health, The University of Sydney, Sydney, NSW, Australia; b Institute of Academic Surgery, Royal Prince Alfred Hospital, Camperdown, NSW, Australia

**Keywords:** Medical education, surgical education, surgical training, surgical residency, intraoperative learning

## Abstract

This study aims to understand the perspectives of surgeons and trainees on intraoperative surgical education, identifying perceived barriers and facilitators. Surgical training is shifting towards competency-based medical education (CBME) with the goal of graduating measurably competent surgeons. Despite this, concerns remain that trainees lack the clinical exposure to develop surgical competency. Understanding surgeon and trainee perspectives on intraoperative education can help identify current issues and provide direction for improving surgical training. A systematic review and meta-synthesis of qualitative studies that assess surgeon and trainee perspectives of intraoperative education was performed. 11,287 papers were screened, and 60 met the inclusion criteria, representing the perspectives of 1592 surgeons and trainees. Three major themes emerged that emphasised (1) the importance of developing trust, (2) the key qualities of effective surgeon educators, and (3) the impact of financial incentives, medico-legal concerns and educational support in the educational environment. This review provides a framework to understand intraoperative education and identifies key facilitators and barriers for surgeons, trainees and training programs.

## Introduction

Historically, surgical training has relied on a time-based apprenticeship model between surgeons and trainees. By the end, trainees are expected to graduate as competent surgeons; however, concerns have arisen with the changing landscape of modernised healthcare [1–6]. Modern healthcare reforms have improved patient care, hospital efficiency, and trainee well-being [7]. However, heightened litigation, progressive privatisation, and duty-hour restrictions have potentially reduced educational opportunities [4,^5^,8]. There are concerns that surgical trainees may be graduating without the required competency for surgical practice, suggesting a need for improved education and assessment of competencies [2,^6^,^9^,10]. Surgical colleges globally have responded by implementing competency-based medical education (CBME), utilising workplace-based assessments (WBAs) to link competency to practical tasks performed during training [11–13]. Simulation-based training has also been suggested as a supplement or alternative to intraoperative education [14,15]. However, assessing competency has proved challenging practically, and despite the potential of simulation, it is still not widely incorporated into training programs [14–18]. Thus, surgical programs continue to rely on time-based training and intraoperative education [14,^15^,^19^,20]. Understanding how intraoperative education occurs is essential for both assessment and education of competency in modern surgical training. Understanding the perspectives of surgeons and trainees can help define how this learning takes place, identify the barriers and facilitators, and reveal ways to improve the quality of education.

The primary aim of this study was to synthesise the literature on surgeon and trainee perspectives of intraoperative education. The secondary aim was to develop a conceptual framework to understand how intraoperative education occurs and identify facilitators or barriers to education.

## Materials and methods

Enhancing transparency in reporting the synthesis of qualitative research (ENTREQ) guidelines were followed in the design and reporting of this review [21]. It was registered with the international prospective register of systematic reviews (PROSPERO) on the 26^th^ of February 2024 in accordance with the PRISMA-P guidelines (PROSPERO:CRD42024513361) [22].

### Eligibility criteria

To be eligible for inclusion, studies needed to explore surgeon and/or trainee perspectives of intraoperative education. Studies utilising qualitative methods for collecting and analysing data were included. Mixed-method studies with extractable qualitative components were also included. All surgical specialties were included. Only original articles were included. Studies were excluded if they were: non-English, not peer-reviewed, conference abstracts, opinion-pieces, or reviews. Studies were also excluded if they focused on medical students, veterinarian surgery, or simulation training only.

### Search strategy

An online database search was conducted through Ovid Medline, Ovid Embase, Web of Science, and Google Scholar (first 200 articles) with university librarian assistance. Four key concepts (1) ‘intraoperative,’ (2) ‘education,’ (3) ‘surgeon trainer,’ OR ‘surgical trainees’ and (4) ‘perceptions’ were defined. These key concepts and associated subject headings were searched with ‘OR’ and then combined with ‘AND’ (see the Appendix for full database search strategy). The search included all studies up to 2^nd^ June 2025.

### Study selection process

**Figure 1.**
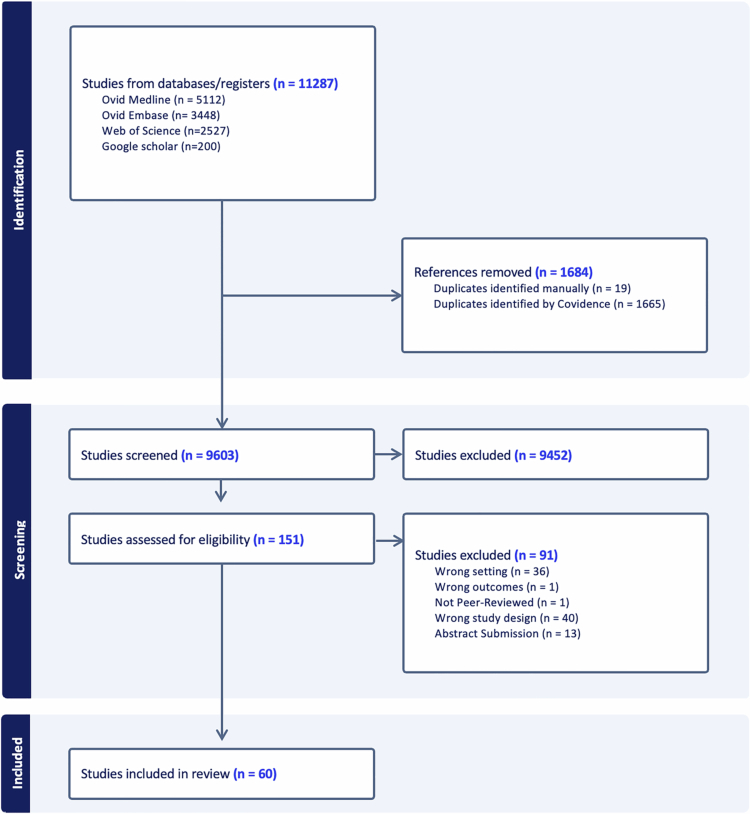
PRISMA flow diagram of study identification, screening and inclusion (Covidence) [23].

The search results were imported into Covidence online software [23]. Duplicate and non-English studies were removed. Title and abstract screening began with two researchers for the first 10% of the papers, at which point consistency in the criteria was reached, and the remaining studies were screened by one researcher (IE). The full text of 133 papers were screened by two researchers for the first 10% and one researcher (IE) for the remainder. Any disagreement was resolved as a group (IE, RF, SW and JH). See Figure 1 for the selection process.

### Data extraction and meta-synthesis

Qualitative data from individual studies was independently extracted by one researcher (IE) and reviewed by our team (RF, SW and JH) to ensure reliability. Qualitative data was then coded into surgeon and trainee perceptions of intraoperative learning following the approach to thematic analysis outlined by Braun and Clarke [24]. Codes were grouped into sub-themes. Sub-themes were used to generate the main themes. The meta-synthesis of new themes was reached by team consensus and helped to conceptualise a framework for intraoperative education.

### Quality assessment

The Joanna Briggs (JB) Institute checklist for qualitative research bias [25] was used to assess the quality of each study.

### Reflexivity

Our team consisted of a surgical trainee (IE), surgeons (JH and JL), a nurse educator (SW), and a health sociologist with expertise in qualitative research (RF). Diverse perspectives offered both first-hand experience, as well as a broad interpretation of themes to mitigate researcher bias.

## Results

Among the 11,287 studies identified in our literature search, a total of 60 met our criteria for inclusion.

### Study characteristics

See Table 1 for a full list of included studies and their characteristics. The 60 studies included represented the perspectives of 1,592 surgeons and trainees. Sixty-two percent of the studies were conducted in the USA. Eighty-five percent of the studies were conducted in the last 10 years. The vast majority used interviews or focus groups (> 90%). All surgical specialities were included, and general surgery was most prevalent (33/60) followed by mixed specialties (16/60) and obstetrics and gynaecology (6/60).

**Table 1.** Study characteristics of the included studies and JB quality assessment score.

Author (year)	Country	Setting	Surgical specialty	Data collection	Participants	JB (/10)
**Abahuje (2023)** [26]	USA	15 Surgical residency programs	General	Interviews, focus groups and surveys	54 (Unspecified number of surgeons & trainees)	6
**Ahle (2020)** [27]	USA	National surgical residency program	General	Interviews	20 Surgeons	7
**Anderson (2023)** [28]	USA	15 Surgical residency programs	Mixed specialties	Interviews	31 Left-handed trainees	9
**Apramian (2016)** [29]	Canada	3 Academic hospitals	(Not specified)	Interviews and observations	56 Operations	8
**Apramian (2016)** [30]	Canada	3 Academic hospitals	(Not specified)	Interviews and observations	14 Surgeons	8
**Brooks (2023)** [31]	USA	4 Academic hospitals	General	Interviews	13 Surgeons and 6 trainees	8
**Carter (2023)** [32]	UK	1 Academic hospital	General, orthopaedics and urology	Interviews	8 Surgeons, 8 trainees and 8 surgical assistants	2
**Cassidy (2021)** [33]	USA	1 Surgical residency program	General	Focus groups	20 Trainees	5
**Chen (2015)** [34]	USA	1 Academic hospital	General	Interviews and observations	3 Surgeons	6
**Chen (2016)** [35]	USA	3 Academic hospitals	O&G	Interviews	13 Trainees	0
**Chen (2019)** [36]	USA	21 Academic hospitals	General and O&G	Interviews	43 Surgeons	4
**Collings (2023)** [37]	USA	1 Surgical residency program	General	Focus groups	39 Trainees	9
**Colon-Lopez (2024)** [38]	USA	1 Academic hospital	General	Interviews and observations	17 Surgeons and 10 trainees	2
**Cooper (2022)** [39]	UK	1 Academic hospital	Plastic	Interviews	5 Surgeons and 5 trainees	3
**Dath (2013)** [40]	Canada	1 Academic hospital	Mixed specialties	Focus groups	44 Surgeons	4
**Debono (2023)** [41]	France	National surgical residency program	Neurosurgery	Interviews	23 Trainees	7
**Devries (2021)** [42]	USA	4 Surgical residency programs	General and otolaryngology	Interviews	14 Trainees	6
**Dickinson (2020)** [43]	USA	1 Surgical residency program	General	Interviews and surveys	13 Trainees	6
**Doster (2022)** [44]	USA	1 Academic hospital	General	Focus groups	39 Trainees	6
**Fieber (2021)** [45]	USA	1 Surgical residency program	General	Interviews	10 Surgeons and 13 trainees	4
**Geary (2023)** [46]	USA	4 Surgical residency programs	General	Interviews	10 Surgeons and 11 trainees	4
**Go (2024)** [47]	USA	1 Surgical residency	General	Interviews	5 Surgeons and 5 trainees	5
**Hammond (2020)** [48]	Canada	1 Surgical residency program	General	Interviews	10 Surgeons and 12 trainees	8
**Heidt (2023)** [49]	USA	1 Academic hospital	General	Surveys	11 Surgeons and 19 trainees	2
**Hill (2017)** [50]	USA	1 Surgical residency program	General	Interviews	8 Surgeons and 9 trainees	4
**Kaba (2025)** [51]	USA	National surgical residency program	Urology	Interviews	20 Trainees	6
**Kamali (2018)** [52]	UK	1 Surgical residency program	General	Interviews	15 Trainees	8
**Kieu (2015)** [53]	Australia	1 Academic hospital	General	Interviews	10 Surgeons and 10 trainees	5
**Knifed (2010)** [54]	Canada	1 Surgical residency program	Mixed specialties	Interviews	28 Trainees	5
**Lambert (2025)** [55]	Netherlands	1 Academic and 5 teaching hospitals	General and orthopaedic	Interviews and observations	16 Surgeons and 16 trainees	7
**Moulton (2010)** [56]	Canada	4 Academic hospitals	General	Interviews	28 Surgeons	7
**Ong (2015)** [57]	Singapore	1 Surgical residency program	Mixed specialties	Interviews and observations	5 Surgeons and 5 trainees	10
**Othman (2022)** [58]	Australia	1 Academic hospital	Mixed specialies	Interviews	2 Surgeons and 3 trainees	5
**Ott (2018)** [59]	Canada	2 Academic hospitals	General	Interviews and observations	9 Surgeons and 9 trainees	8
**Papachristos (2021)** [60]	Australia	National surgical residency program	General	Interviews	10 Trainees	7
**Papachristos (2024)** [61]	Australia	1 Academic hospital	General	Interviews	11 Trainees	8
**Pernar (2012)** [62]	USA	National surgical residency program	General	Surveys	21 Surgeons	3
**Ranney (2021)** [63]	USA	1 Academic hospital	General	Interviews and focus groups	2 Surgeons and 14 trainees	7
**Rivard (2022)** [64]	USA	1 Academic Hospital	Mixed specialties	Feedback forms	14 Surgeons and 46 trainees	6
**Sadati (2021)** [65]	Iran	National surgical residency program	General	Interviews and observations	5 Surgeons and 25 trainees	4
**Sadati (2021)**[66]	Iran	11 Academic hospitals	General	Interviews	25 Trainees	7
**Salim (2020)** [67]	Canada	1 Surgical residency program	General	Interviews	9 Surgeons	7
**Sampene (2019)** [68]	USA	1 Academic hospital	O&G	Interviews and observations	5 Surgeons and 5 trainees	5
**Sandhu (2017)** [69]	USA	41 Academic hospitals	General	Interviews	37 Surgeons and 59 trainees	6
**Singh (2014)** [70]	UK	3 Surgical residency programs	General	Interviews	10 Surgeons and 10 trainees	5
**Smith (2021)** [71]	USA	1 Surgical residency program	O&G	Focus groups	20 Trainees	4
**Smith (2017)** [72]	USA	2 Academic hospitals	Mixed Specialties	Interviews	23 Surgeons	6
**Smith (2019)** [73]	USA	1 Surgical residency program	General, O&G and urology	Focus groups	25 Trainees	7
**Sutkin (2020)** [74]	USA	1 Academic hospital	O&G	Interviews and observations	5 Surgeons and 5 trainees	5
**Sutkin (2019)** [75]	USA	1 Academic hospital	O&G	Interviews and observations	5 Surgeons and 5 trainees	5
**Swendiman (2019)** [76]	USA	1 Surgical residency program	General, plastics and vascular	Interviews	12 Surgeons	5
**Teman (2014)** [77]	USA	7 Academic hospitals	(Not specified)	Surveys	116 Surgeons	4
**Torbeck (2015)** [78]	USA	1 Academic hospital	General	Interviews and focus groups	10 Surgeons and 20 senior trainees	8
**van der Houwen (2011)** [79]	Netherlands	4 Surgical residency programs (32 hospitals)	O&G	Focus groups	24 Surgeons and 32 trainees	7
**Vikis (2008)** [80]	Canada	1 Surgical residency program	General	Interviews	18 Trainees	3
**Vollmer (2011)**[81]	USA	5 Academic hospitals	General	Surveys	37 Surgeons and 46 trainees	5
**Winer (2024)** [82]	USA	16 Surgical residency programs	General	Surveys	96 Trainees	3
**Woelfel (2020)**[83]	USA	2 Surgical residency programs	General and O&G	Focus groups	38 Trainees	4
**Woelfel (2023)**[84]	USA	8 Academic hospitals	Mixed specialties	Interviews	23 Surgeons	6
**Zmijewski (2025)** [85]	USA	National surgical residency program	General	Focus groups	22 Trainees	7

JB, Joanna Briggs; O&G, obstetrics and gynaecology; USA, United States of America; UK, United Kingdom.

### Quality assessment

A quality assessment was performed using the peer-reviewed JB checklist for qualitative systematic reviews (see supplementary material). The research methodology stated by most papers was thematic analysis (either grounded theory, inductive or deductive). According to our quality analysis (Table 1), this was consistent with the stated research question in 41/60 papers. The methodology was congruent with the methods used, representation, and analysis of data in the vast majority of the papers (50/60 and 49/60). Congruity between the interpretation of the results and the stated methodology was observed in 38/60 papers. Only 17/60 papers showed reflexivity by identifying the researcher culturally or theoretically, and only 4/60 researchers discussed their influence on the research. A total of 47/60 papers included an ethics approval, although few discussed the ethical implications of their research.

### Perspectives on intraoperative education – a framework for intraoperative education

**Figure 2.**
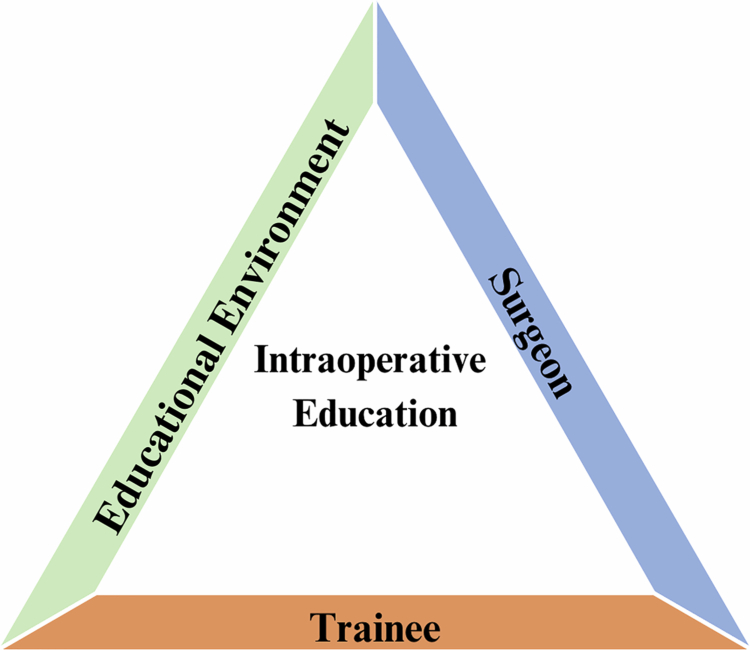
Framework for conceptualising intraoperative education. An apprenticeship model of intraoperative education occurs through the delivery of patient care and is influenced by the relationship between surgeon, trainee, and external factors (educational culture, time, theatre staff, etc.).

Among the 60 studies included, 447 codes were generated from surgeon and trainee perspectives. From these codes, three themes were identified: trainee development of trust (Table 2), the qualities of effective surgical educators (Table 3), and the impact of the educational environment on surgical education (Table 4). These themes helped to conceptualise our understanding of intraoperative education, which is shown in Figure 2.

**Table 2.** Themes – trainee development of trust.

Theme (codes)	Representative quotes
Building trust	
• Trust is developed over time • Seniority/PGY level	‘Trust is built, it’s not implicitly [given]. You’ve got to build trust with time.’ (Surgeon) [26] ‘Generally, procedures with higher educational potential are for attendant residents. Less educational procedures remain for the rest.’ (Trainee) [65] ‘I operate with Dr. X so much, and he has in his mind a curriculum based on PGY year.’ (Trainee) [42]
• Initiative and confidence	‘if people are more prepared, then, they do get more autonomy.’ (Surgeon) [45] ‘If I have a sense that resident has confidence [they will do more].’ (Surgeon) [78] ‘The cowboy or cowgirl I’m more involved with them to make sure they don’t overstep their capabilities.’ (Surgeon) [78]
Maintaining trust	
• Able to demonstrate skill and progress safely	‘If you can do it well and the educator understands that you are capable and you deserve it, [they] will hold your hand, and you will grow.’ (Trainee) [65] ‘They all had to show that to me at least that they are safe. And when I feel they are safe, I let them do a lot.’ (Surgeon) [78] ‘I think it’s a good way for him to still feel comfortable with me operating so I can demonstrate to him that I know what is unsafe territory, what I should be above.’ (Trainee) [74]
• Communicates decision making • Follows instructions	‘They loved that I verbalized every single thing I was doing constantly. Really, as I started verbalizing a ton, I felt like I got to do a ton more. And I was allowed to struggle through things.’ (Trainee) [63] ‘I'm more likely to let her struggle. Especially because she takes instruction well.’ (Surgeon) [50] ‘If you’re a bit too slow, don’t do the right gesture, or you don’t do exactly what they had in mind, etc., then they’ll take over.’ (Trainee) [41]
Barriers to trust	
• Gender • Left-handedness	‘I’ve certainly been biased. I think that male resident tend to over trust their abilities and be over-confident of their abilities, and female residents tend to be under-confident.’ (Female Surgeon) [38] ‘I don't feel as comfortable as my male co-residents delegating work so that I can go to the OR. I also feel pressured to give cases to others so they get experience, and my male co-residents don't seem to do the same.’ (Trainee) [82] ‘I felt it took them up to one year to master use of the diathermy in their right hand, while they felt their RH counterparts only took three months’. (Trainee) [58]

**Table 3.** Themes – qualities of effective surgical educators.

Theme (codes)	Representative quotes
Attitudes and behaviours	
• Engagement with education	‘they see it as their responsibility to help that person become a good surgeon, or become a good physician.’ (Trainee) [80] ‘I think it’s part of our Hippocratic Oath, is to train the next generation of doctors. And I think when we become doctors and surgeons, we take that as part and parcel of training the next generation. And I think forgetting to train your junior is forgetting your Hippocratic Oath.’ (Surgeon) [70]
• Confidence in their own ability • Calm and supportive demeanour • Patience	‘There’s attendings (surgeons) that I know can solve any problem, anything that we (resident) get into. they give us more autonomy than other.’ (Trainee) [71] ‘I think I have felt more comfortable allowing residents to do more and more over the years, largely because I know that I have the ability to get them out of more and more trouble.’ (Surgeon) [27] ‘[They] have an uncanny ability to make everyone in the room feel relaxed. I find everyone is motivated to work harder and better because of the great approach [they] have.’ (Trainee) [64] ‘I genuinely believe the stress [they] induce on all members of the operative team makes any attempts at teaching negligible. The environment is unfortunately not conducive to learning.’ (trainee) [64] ‘[Surgeons] need a lot of patience, because you have to explain everything - of course it increases operating time by 1.5 or even twofold … but, if you are able to do it, then the trainee will leave thinking “wow, that was incredible!”.’ (Trainee) [41]
Teaching techniques (and communication)	
• Allow meaningful responsibility and graduated autonomy • Encourage trainee decision making • Use guiding practice (physical or verbal guidance) • Allow ‘safe-struggle’ (trainees are challenged in a safe setting) • Adapt to trainee’s ability • Technique variation (short-term challenge, long-term benefits) • Teaching assistant (TA) cases • Clear communication	‘shifting the responsibility to the learner as the learner progresses.’ (Surgeon) [62] ‘. (I) gradually get to do more complicated parts of [thyroid surgery] and then I am doing it by myself with supervision - I have done a lot of watching, but at this point I do need to do it.’ (Trainee) [42] ‘Dr. Y did a very good thing that no one else did. before he goes into the OR he talks to the senior resident and he says, “what is your game-plan-. what is your plan of attack?”.’(Trainee) [80] ‘The way he teaches is the way of making me learn, but not of feeling stupid at the same time. He does correct me if I do things wrong, but he does not completely take over it, so I still feel in control of the whole situation.’ (Trainee)[57] ‘The faculty should allow us to struggle a bit more than they do.’ (Trainee) [81] ‘My philosophy is, if they do something wrong they’re done – They don’t have to do something catastrophically wrong, just any wrong move, and I take the case from them.’ (Surgeon) [69] ‘Surgeons who will not take over as soon as I get into trouble – I think that's when I learned the best. When they just pushed me that little bit. But under supervision. Then you realize, oh. actually I can do this. Now that they've made me do this, I feel a little more confident.’ (Trainee) [60] ‘I think the most important thing about teaching is developing an awareness of where the skill set and knowledge of a particular resident is, and then finding a way to progress and improve those skills and knowledge in a stepwise fashion.’ (Surgeon) [81] ‘I don’t care whether she [trainee] is able to choose the right instrument, I just want her to do what I want.’ (Surgeon) [34] ‘If you got taught the same way by every staff, you wouldn’t become a well-rounded surgeon; you need the differences in styles.’ (Trainee) [37] ‘Teaching other residents (was helpful) and I didn’t know how much that taught me until I began (to talk) about it.’ (Trainee) [42] ‘The precision and language [needed] when you’re trying to instruct someone what to do and how you convey that language is critical to maintaining comfort of learner and the operating room and making sure that they understand.’ (Surgeon) [46]

**Table 4.** Themes – impact of the educational environment on surgical education.

Theme (codes)	Representative quotes
Structural barriers	
• Financial incentives	‘The hospital head and even the ministry expect that the public hospital should make money, so there is a need for a great number of operations to generate income. This decreases the quality of operation and education.’ (Trainee) [66] ‘If I’m (surgeon) gonna (slow down to) teach more and my dashboard shows that I can only (schedule to) do two cases a day instead of three, the RVU was out of my pocket that I didn’t generate. It does affect me personally, the more (cases) I do, the more bonus I’m gonna get at the end of the year.’ (Surgeon) [84]
• Hospital and departmental support for teaching • Medico-legal concerns • Scheduling (case volume and exposure)	‘I didn’t realise that it depends on the hospital … in (City B) for example there’s not really the same … teaching philosophy … so, uh … I was a bit surprised to find out that … well … I wouldn’t be operating at all at the beginning of my residency: I was only allowed to watch.’ (Trainee) [41] ‘It would really help the registrars (if there were more structure to intraoperative education) because we're really learning on our own, ad hoc things from here, there and everywhere but there's no system. There's no system to this madness.’ (trainee) [60] ‘Surgeons are less trained….I think everybody acknowledges that one of the elements of the problem…[is] the need for additional supervision because of different medical legal expectations.’ (Surgeon) [26] ‘The current supervision requirements are by far the greatest challenge, and they come from multiple directions e ACGME, JCAHO, patients, insurers, etc. The ability to really let residents develop their autonomy in the operating room with the pressures of being at the table from pre-incision to end and to be increasingly efficient all jeopardize resident development. This graduated responsibility has to be built back into training, but will have to be reconfigured amidst current regulations and patient expectations.’ (Surgeon) [69] ‘There must be workload. It doesn’t have to be excessive. In fact, excessive workload is probably a mistake because people get too busy and then it doesn’t provide a training opportunity.’ (Trainee) [26] ‘The learner must work through enough cases so [they] will have encountered the full spectrum of contextual variety.’ (Trainer) [62])
Competition for educational time	
• Administrative (non-clinical) tasks • Impact of other trainees, fellows or surgical assistants • Time pressure from anesthetics and nursing staff	‘because of, well … the huge administrative burden and myriad tasks to be carried out … which kind of compromises the smooth running of the teaching process.’ (Trainee) [41] ‘[as] the only resident there, I got to scrub for all the hysterectomies. Versus here there’s a lot, a lot more residents (and thus fewer opportunities).’ (Trainee) [71] ‘The [Surgical Assistant] and my interests have clashed and he [was] acquiring his basic surgical skills before [me].’ (Trainee) [32] ‘‘When the theatre team are moaning and groaning because the operation is taking too long – I don’t know how to put it but it makes you feel uncomfortable when you are operating.’ (trainee) [79] ‘I also have to make sure the OR staff is comfortable. And the anaesthesiologists are comfortable.’ (Surgeon) [46]
Patient factors and unpredictable nature of surgery	
• Case uncertainty • Patient expectations • Awake patients	‘Unexpected findings basically or changing the operative plan within the case [put a pause on teaching].’ (Surgeon) [46] ‘Patient expectation that the Attending is the operating surgeon (reduces teaching).’ (trainee)(Vollmer) You want to rule out those people that are husbands or wives of surgeons, because they’re generally pretty savvy, and they don’t want to be operated on by a resident.’ (Surgeon) [72] ‘it’s a lot harder to sort of show a trainee a procedure when a patient’s watching, right? Because then the patient really sees that someone else is doing it, which is sometimes a little bit upsetting to people.’ (Surgeon) [72]

### Trainee development of trust

Three main sub-themes emerged relating to trainee development of trust: building trust with their supervising surgeon, maintaining trust intraoperatively, and overcoming barriers to trust (Table 2).

### Building trust

Trainees’ ability to build trust is considered the foremost consideration for surgeons facilitating intraoperative teaching whilst maintaining patient safety. Trust was developed over time [26–38], however, trainee seniority [28,33–35,39–45], initiative and confidence [26,28–35,37,42–44,46–61] were seen as crucial in building this trust. Competency-based programs could potentially accelerate trust formation and graduated autonomy [37]. Trainees’ confidence in their decision-making is often perceived as beneficial for entrustment [26,28,50]. However, ‘over-confidence’ was equated with being unsafe and this hindered autonomy [26,37].

### Maintaining trust intraoperatively

Trainees who would operate efficiently and progress the operation, whilst also demonstrating principles of patient safety, maintained surgeons’ trust for longer [26,30,31,33,34,41,45,47,48,54,57,60,62–64]. Slowing for important steps, verbalising concerns, and being reflective are ways of demonstrating these principles to surgeons [34,45,50,64,65]. Surgeons were more willing to allow trainees to have control of a case if the trainee could follow verbal or physical instructions [26,28,30,31,47,50,57,60,61,66]. Trainees communicating their decision-making also improved intraoperative education [28,40,46,47,49,52].

### Barriers to trust

In certain contexts, trust for female [33,35,37,38,41,67] and left-handed [68–70] trainees was considered harder to develop and maintain. Female trainees in Iran felt that they received less support and intraoperative education than their male colleagues [67]. In the USA, some surgeons have suggested that an unconscious bias towards male trainees affecting entrustment decisions [35], whilst female trainees themselves felt less confident in theatres, and have greater administrative burdens than male colleagues [37,38]. Left-handed trainees described initial difficulties maintaining intraoperative trust whilst adjusting to right-handed instruments [68–70]. However, these issues lessened as they developed ambidexterity [69].

### Qualities of effective surgeon educators

Two sub-themes emerged relating to the qualities of effective surgeon educators, which could be categorised as attitudes and behaviour, and teaching techniques (Table 3).

### Attitudes and behaviours

Surgeons who were engaged in education who were calm, and portrayed confidence in their own ability were perceived to enhance intraoperative education. Engagement in education was motivated by a sense of responsibility or enjoyment [26,27,32,48,53,71]. Whilst surgeons who disliked teaching, or considered teaching a risk to patient safety were seen as less effective educators [26,28,30,39,42,44,47,49,57,60,72]. Surgeons demonstrating a supportive and calm demeanour were valued by trainees and perceived to improve learning [28,52,62,66,71,73,74]. However, intimidating and volatile behaviour was perceived to ‘make any attempts at teaching negligible’ (Trainee) [57] and reduce trainee confidence [29,52,66,74,75]. Patience was perceived as necessary for creating the time and space for education to occur and was critical to guided-practice and safe-struggle [26,31,46,47,49,52,57,58,73]. Finally, surgeon self-confidence is perceived to facilitate progressive autonomy and trainee operative learning [29,34,40,43–45,47,49,58,60,64,71,75,73,76].

### Teaching techniques (and communication)

Surgeon teaching techniques and communication style were perceived to greatly influence intraoperative education. Surgeons who granted trainees meaningful responsibility through graduated operative autonomy [26–28,31,32,37,40,42,43,49,51–53,56,58,60–62] and decision-making were highly valued [26–28,30–32,40,42,43,46,48,49,51–60,62,71,72,74]. One study describes that giving trainees ‘direct control’ (primary operator role) was dependent on surgeons maintaining ‘overall control’ [30]. Effective educators maintained this control through guided-practice (verbal or physical) [26,28,29,49,51,53,54,57,63,72,77] and were able to provide trainees with opportunities for ‘safe struggle’, which trainees felt critical to progression [26,28,30,43,46,47,49,52,58,72]. An adaptable teaching approach based on trainee ability, not PGY level was valued [27,29,46,49,66,73,74,78]. Surgeon operative technique variations initially challenged trainees [64]; however, in the long term, ‘you wouldn’t become a well-rounded surgeon [without learning variations]’ (trainee) [74]. Surgeons who encouraged teaching assistant (TA) cases, where the senior trainee guides a more junior trainee through an operation, were valued [26,31,35,40,43,44,50,54,56,72,79,80]. With TA cases, trainees can learn additional technical skills (e.g., retracting for guidance) and cognitive skills (communication, teaching, and organisation) [56]. Finally, clear communication from surgeons was perceived to improve education, help trainees follow instructions, and progress operative skills [42,56,58,62,73–75].

### Impact of the educational environment on surgical education

Three sub-themes describe the impact of the educational environment on intraoperative education: structural barriers, competition for educational time, and patient factors (Table 4).

### Structural barriers

Financial incentives, teaching culture, medico-legal concerns, and list scheduling were seen to impact intraoperative learning. Financial incentives, such as private lists, maximising relative value units (RVUs) and the payment structure were seen as incentives to book lists fully, leaving less time for teaching [26,32,39,41,46]. Whilst salary-based surgeons feel less financial pressure and more time to teach [39]. Hospital and departmental support for teaching was reported to improve intraoperative education [26,27,29,31,36,42,44,49,54,61,67,70,71,73,78,81], ‘good teaching isn’t something that will happen without a department-wide dedication to making it a priority’ (surgeon) [49]. A heightened concern for potential medico-legal complaints was seen to be an ongoing issue facing education [26,32,35,45,46,49,56,67,78], with one surgeon postulating ‘increased legal pressures would soon be an insurmountable barrier to allowing teaching assistant (TA) cases without legislative intervention’ (surgeon) [56]. Finally, a reasonable case-volume and exposure to the ‘full spectrum of contextual variety’ (surgeon) [51] was considered to improve intraoperative learning [27,40,51,71,72,78].

### Competition for educational time

Competition for educational time came from administrative (non-clinical) tasks, other trainees or fellows, and non-surgical theatre staff (anaesthetics and nursing). Intraoperative education required time, and non-operative, or ‘administrative’ duties were felt to reduce trainee theatre time [31,32,37,38,42,49,52,62], for example, ‘being distracted by pages from the floor, or consults’ (surgeon) [49]. Fellows [67], surgical assistants (SAs) [82] or even other trainees [40] were perceived as potential competition for intraoperative learning by trainees. However, this impact appeared to be context-dependent with some perceiving SAs and fellows to improve operative learning [67,82]. Anaesthetics and nursing staff put pressure on surgeons to operate to expedite a list, particularly towards the end of the day [47,56].

### Patient factors and unpredictable nature of surgery

Unforeseen circumstances, such as rapid changes in the patient’s clinical status, patient expectations, or awake operations, could impact the educational experience. Case uncertainty may diminish trainee autonomy [29,34,54,56,57,74], yet trainees reflected that ‘autonomy can vary based on case complexity and that is ok’ (Trainee) [74]. Surgeons felt that they can still find teaching opportunities in these instances [71]. Patients’ expectation to have the surgeon perform their operation was perceived to reduce trainee involvement [40,80,83,84]. For awake operations, or where patients knew the team personally or were healthcare workers, this was more common [40,83,84].

## Discussion

This systematic review synthesised the perspectives of 1,592 surgeons and trainees across 60 studies to understand intraoperative education, identifying potential facilitators and barriers. Three key themes emerged: (1) trainee development of trust, highlighting how trust is built and maintained between trainees and supervising surgeons; (2) qualities of effective surgical educators, describing the attitudes, behaviours, and teaching techniques that enhance learning; and (3) impact of the educational environment, emphasizing systemic, logistical, and patient-related factors that shape intraoperative teaching. These themes helped conceptualise a framework for intraoperative education (Figure 2).

Our findings reinforce the importance of trust and surgeon–trainee relationship in the literature [46]. Our study showed building trust took time and was improved with trainee seniority, initiative, and confidence. Trends toward competency-based training programs may help improve trust through the use of entrustable professional activities (EPAs) [85]. Once established, trainees need to maintain trust intraoperatively through safe progression and communication. Difficulties for female and left-handed trainees in terms of building and maintaining trust were also identified in our study. Meyerson et al. (2019) showed female trainees receive less autonomy overall (*P* < .001) [86], whilst other quantitative studies have shown no significant difference [87,88], suggesting that these results may be context specific, and understood with further research.

**Figure 3.**
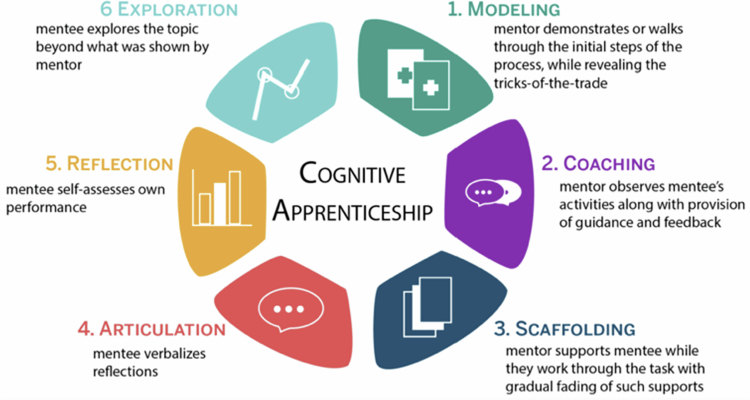
Cognitive apprenticeship model (Kurt 2021) [105]. Describes an experiential ‘apprenticeship’ learning model whereby a trainee develops graduated autonomy within the functioning workplace.

Our study showed that effective surgical educators demonstrated engagement, patience, and an ability to provide progressive autonomy while maintaining patient safety. Teaching techniques such as guided-practice, ‘safe-struggle,’ and adaptive teaching based on skill level (rather than training year) were particularly valued. These findings align with previous research highlighting the benefits and safety of progressive autonomy for trainees [89–95]. This is consistent with the cognitive apprenticeship model (Figure 3), which emphasises the importance of progressive participation (graduated trainee autonomy) [76]. Tools such as ‘educational time-outs’ or workplace-based assessments (i.e., EPAs) may improve engagement with education in the operating room [96–98]. Future research should look at how these tools are being implemented and their impact on intraoperative education.

The educational environment significantly influences intraoperative learning, which aligns with the current literature suggesting financial pressures [8], litigations claims [99,100], and reduced education time [101–103] are growing barriers to training. These barriers may be mitigated by an institutional and departmental commitment to teaching – rather than solely relying on individual surgeon initiative. Recent research suggests that recognition and financial incentives for teaching may help overcome some of these barriers [104]. Ultimately, education needs to be prioritised on an organisational level.

A key strength of this study is its comprehensive synthesis of qualitative perspectives across multiple surgical specialties, providing a broad, yet detailed understanding of intraoperative education. The quality assessment of studies demonstrated overall methodological congruency; however, the limited researcher reflexivity and the predominance of studies conducted in the USA, may limit generalisability.

## Conclusion

This review provides a framework to understand how intraoperative education occurs and identifies key facilitators and barriers. For trainees, the importance of building and maintaining trust is highlighted. For surgeons, facilitating the graduated autonomy of their trainees whilst maintaining a calm demeanour and clear communication was highly valuable. Our framework also helps conceptualise the current structural barriers to intraoperative education, suggesting that financial, medico-legal and time constraints need to be addressed to optimise modern surgical training. Future studies should address these structural barriers, and interventions should focus on improving the surgeon–trainee relationship whilst empowering trainee progressive autonomy.
